# A Case of Chronic Pulmonary Embolism Resulting in Pulmonary Hypertension and Decompensated Right Heart Failure

**DOI:** 10.7759/cureus.32771

**Published:** 2022-12-21

**Authors:** Asma U Hosna, Daniel Miller, Karim Makhoul, Nicole Noff

**Affiliations:** 1 Internal Medicine, Icahn School of Medicine at Mount Sinai, Queens Hospital Center, New York, USA

**Keywords:** medical management, right heart failure, right heart strain, pulmonary endarterectomy, bilateral pulmonary thromboembolism

## Abstract

Chronic thromboembolic pulmonary hypertension is a deadly condition that results from thrombus organization and formation of fibrous tissue in the large and/or middle-sized pulmonary artery; as a result, pulmonary vascular resistance increases resulting in pulmonary hypertension and right heart failure. Untreated chronic pulmonary embolism causes decompensated right heart failure. Early diagnosis and appropriate treatment are crucial for improving survival. Pulmonary endarterectomy (PEA) is the treatment of choice as it reduces pulmonary vascular resistance. For patients who are not a candidate for PEA, alternative treatment options improve quality of life.

## Introduction

Chronic thromboembolic pulmonary disease (CTEPD) is a term used to describe patients who present with shortness of breath, and imaging shows findings of chronic organized clots suggestive of chronic thromboembolism. A ventilation/perfusion (V/Q) scan shows mismatched perfusion defects. Computed tomographic pulmonary angiography (CTPA) shows clots in the pulmonary artery after three months of therapeutic anticoagulation therapy. Ring-like stenosis, webs, slits, and chronic total occlusion are the specific diagnostic signs of CTEPD which are appreciated on CTPA [1,2]. Presentation of pulmonary embolism (PE) can vary from chronic exertional dyspnea to syncope, hemoptysis, and lower extremity edema which can be due to acute PE or sub-acute PE or chronic PE [3]. Studies showed that up to 25% of patients with chronic thromboembolic pulmonary hypertension (CTEPH) have no known history of prior PE [4].

CTEPH requires a thorough assessment and evaluation for appropriate management. Surgical intervention which is pulmonary thromboendarterectomy remains the gold standard treatment for CTEPH [5]. Patients who are not a candidate for surgical intervention or who still have signs of residual pulmonary hypertension after surgery may be a candidate for catheter-based intervention with balloon pulmonary angioplasty or medical management [4].

## Case presentation

CTAA case of a 66-year-old man, body weight 61 kg, height 5"10', and BMI 19.2, with a past medical history of chronic obstructive pulmonary disease (COPD), tuberculosis as a child (spent eight months in the hospital), deep vein thrombosis (DVT) and PE on apixaban, polysubstance abuse, and hepatitis C, presented to ED with the complaint of progressive shortness of breath for two months. As per the patient's son, the patient was having shortness of breath with rest and exertion since he was discharged from the hospital. It was so severe that even taking two steps would bring him to his knees with coughing and shortness of breath. His symptoms started in July, he started to have shortness of breath, and his exercise tolerance decreased from a few blocks to one block. In August, he was hospitalized, and since his discharge, he has been having shortness of breath with rest and exertion. He is currently enrolled in a methadone program. The patient denied any other complaints at the presentation.

In the emergency department, the patient was in moderate respiratory distress and aroused easily, respiratory rate was 25 breaths/minute, BP was 175/134 mmHg, rectal temperature was 93 degrees Fahrenheit, and saturation was 70% on room air, with facemask 15 L saturating 90%. Physical examination revealed decreased breath sound on the right lung base, anasarca, and clubbing. There is an accentuated pulmonic component of the second heart sound and a significant right ventricular heave. S1 and S2 were audible without a murmur. A palpable, nontender left parotid mass was noticed. No lymphadenopathy was appreciated. Bedside point-of-care ultrasound (POCUS) showed moderate right-sided pleural effusion, B-lines on the left side, dilated right ventricle (RV), and preserved ejection fraction (EF).

**Figure 1:**
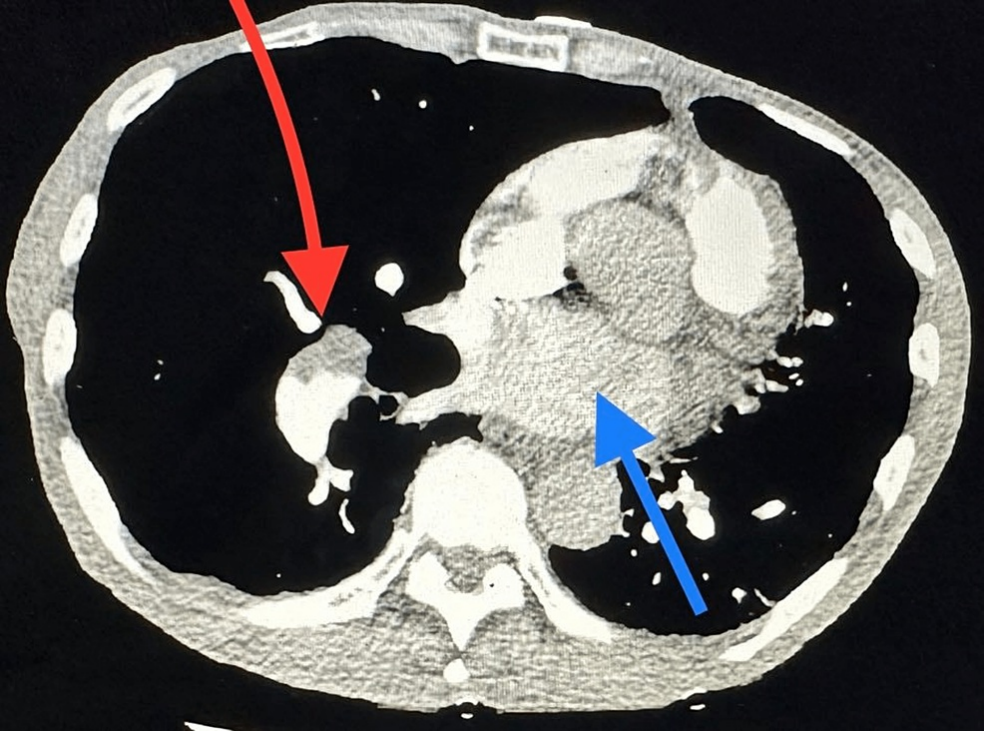
CTA chest showing mural thrombus in the right lung (red arrow) Dilated pulmonary artery (blue arrow) consistent with pulmonary artery hypertension. CTA: computed tomography angiography.

**Figure 2:**
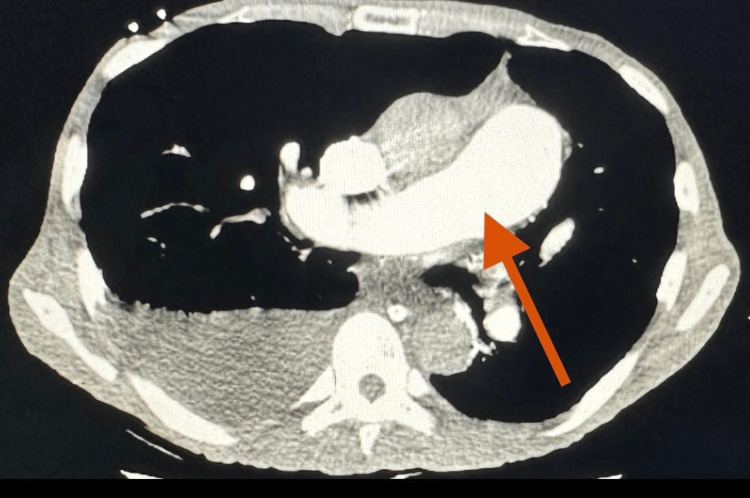
CTA chest showing dilation of pulmonary artery (red arrow) CTA: computed tomography angiography.

Initial laboratory assessment showed no leukocytosis (white blood cell count was 3.91 x 10^3^/µL), erythrocytosis (red blood cell count was 6.44 x 10^6^/µL), hemoglobin of 19.9 g/dl, hematocrit of 64.5%, thrombocytopenia (platelet count was 51 x 10^3^/µL), acute kidney injury with a serum creatinine of 1.37 mg/dl, and respiratory acidosis with pH of 7.31, partial pressure of carbon dioxide (PCO_2_) of 60, partial pressure of oxygen (PO_2_) of 73, lactate of 2.3, bicarbonate (HCO_3_) of 30, hyperkalemia with potassium of 9.8, transaminitis with aspartate aminotransferase (AST) of 86 U/L, and pro-B-type natriuretic peptide (pro-BNP) of 5,730 pg/ml (Table 1). Computed tomography angiography (CTA) chest on admission showed a mural thrombus in the right pulmonary artery extending into the right lower lobe as well as small pulmonary emboli in the anterior right upper lobe (Figures 1, 2). Echocardiogram (ECHO) finding showed mild tricuspid regurgitation (TR), peak aortic valve velocity (Vmax) ~2.9 m/s, pulmonary artery systolic pressure (PASP) was 64 mmHg, severely dilated RV and RV wall hypokinetic, pulmonary artery systolic pressure of 51.00 mmHg, EF was preserved, and central venous pressure was 11-20 mmHg.

**Table 1:** Pertinent lab values on the day of admission and at the time of discharge BUN: blood urea nitrogen, AST: aspartate aminotransferase, Pro-BNP: pro-B-type natriuretic peptide, PCO_2_: partial pressure of carbon dioxide, HCO_3_: bicarbonate.

Lab results (units and reference range)	Day of admission	Day of discharge
Hemoglobin (14.0-18.0 g/dl)	19.9	18.5
White blood cells (4.80-11.80 x 10^3^/µl)	3.91	4.17
Hematocrit %	64.5	58.6
Platelets (150-450 x 10^3^/µl)	51	60
BUN (6-23 mg/dl)	34	29
Creatinine (0.70-1.20 mg/dl)	1.37	1.18
Potassium (3.5-4.1 mmol/L)	9.8	4.4
AST (5-40 U/L)	86	28
Pro-BNP (pg/ml)	5,730	11,293
Venous PCO_2_	73	65
Venous HCO_3_	35	32
Venous pH	7.25	7.40

The patient was started on therapeutic enoxaparin sodium, azithromycin, methylprednisolone, and furosemide. The patient was transferred to Bellevue Hospital Center for evaluation of catheter-directed tissue plasminogen activator (TPA). Upon arrival at Bellevue Hospital Center, the patient was evaluated for TPA. Based on CTA findings, it was concluded that PE was submassive and some appeared chronic. The patient was continued on therapeutic Lovenox which was later switched to direct oral anticoagulant (DOAC). The patient was discharged home on apixaban.

**Figure 3:**
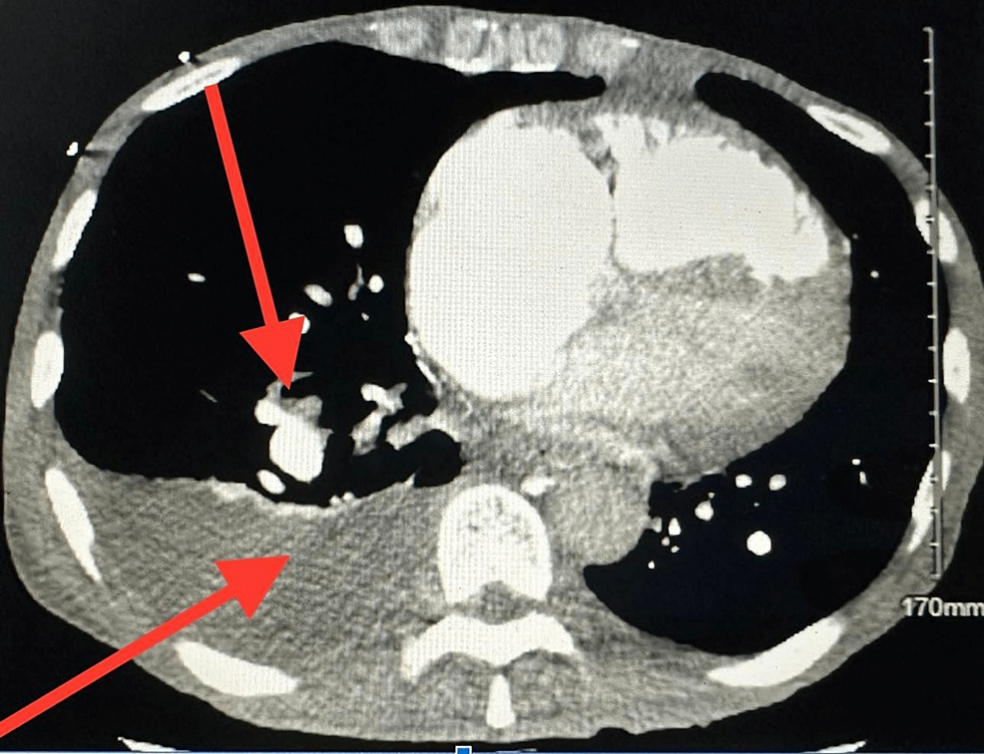
Repeat CTA during readmission showing chronic pulmonary embolus unchanged of descending right interlobar pulmonary artery (red arrows) Small right-sided pleural effusion. CTA: computed tomography angiography.

But after two months, the patient again presented to the Queens Hospital Center (QHC) Emergency Department with shortness of breath. The patient was non-compliant with medications. CTA chest on this admission showed chronic pulmonary embolism of the descending right interlobar pulmonary artery and pulmonary arterial hypertension with evidence of right heart strain with small right-sided pleural effusion (Figure 3).

The patient was started on methylprednisolone, ipratropium-albuterol, budesonide-formoterol, and azithromycin. He was initially tried on a high-flow nasal cannula (HFNC) and was able to maintain his saturation well. Bedside POCUS showed moderate right pleural effusion, B-Lines on the left side, dilated RV, and preserved EF with windows limited due to body habitus. Heme was consulted for this patient for erythrocytosis and thrombocytopenia. Heparin-induced thrombocytopenia (HIT) anti-heparin antibodies and the serotonin-releasing assay were negative. Repeat labs with elevated RBC and hemoglobin (HBG) with normal erythropoietin, negative JAK2 V617F mutation, negative JAK2 exons 12-15, and iron studies within normal limits were observed. The conclusion was made that polycythemia was secondary to chronic hypoxia. The patient had an echocardiogram which showed a severely dilated right ventricle, the right atrium is also dilated, the right ventricle is hypokinetic, and the estimated PASP is 64 mmHg. The paradoxical motion of the interventricular septum and the change of the interventricular septum are consistent with right ventricular pressure overload. Lower extremity venous duplex showed left middle femoral vein, left distal femoral vein, left popliteal vein non-occlusive deep vein thrombosis, and left gastrocnemius vein acute occlusive deep vein thrombosis.

On the second day, his mental status deteriorated (alert, awake, oriented (AAO) x 0, became drowsy), and ICU was consulted for further management. The patient was upgraded to ICU; initially, he was on bilevel-positive airway pressure which was later switched to a high-flow nasal cannula at a rate of 30 ml/hr and 40% O_2_ with nightly bilevel-positive airway pressure (BIPAP). Apixaban was continued in the setting of a history of recurrent PE. Furosemide was started at 80 mg twice daily for a goal net negative at least 1 L fluid balance to improve respiratory status. The patient was treated with cefepime, azithromycin, and vancomycin for sepsis secondary to a urinary tract infection. Later on, piperacillin and tazobactam were started to ensure broad-spectrum microbial coverage and for aspiration pneumonia. The patient was maintained on BIPAP and a high-flow nasal cannula with a low threshold for intubation. As the patient was stable hemodynamically and did not require intubation, the patient was downgraded to the step-down unit. The patient signed out against medical advice, but later that day, the patient was readmitted for shortness of breath and a syncopal episode. CT head negative for acute intracranial pathology or ischemia. The patient kept refusing BIPAP, but he was maintaining saturation to the mid-90s on a high-flow nasal cannula and was compliant with this modality. Serial arterial blood gas (ABG) demonstrated slight alkalosis with mild but much-improved hypercapnia compared to the initial presentation at admission. Even though his pro-BNP trended up, his clinical condition has improved significantly. The patient was discharged home with apixaban 5 mg twice daily and a home O_2_ 4 L nasal cannula. As the patient was non-compliant with medication and constantly using crack, the patient was not a candidate for surgical evaluation and he was referred to the CTEPH clinic for further evaluation.

## Discussion

CTEPH is classified as group 4 pulmonary hypertension, but it differs from other forms of pulmonary hypertension because, in CTEPH, thrombotic material remains in the pulmonary artery for more than three months even after treatment and by the presence of mismatched segmental defects on the ventilation/perfusion scan in CTEPH [6]. Pulmonary emboli can arise due to preexistent deep vein thrombosis or clotting disorder, ventriculoatrial shunts, infected pacemakers, splenectomy, cancer, infections, and thyroid replacement therapy [1]. If it does not resolve after treatment, CTEPH results in the formation of fibrous tissue and causes obstruction of pulmonary vessels resulting in increased pulmonary vascular resistance and right heart failure.

Untreated CTEPH has a very poor prognosis and a high mortality rate. Early diagnosis of CTEPH is a big challenge as the clinical presentation is non-specific and lacks specific guidelines regarding screening people after PE for CTEPH [7]. Even though the development of CTEPH is rare after 24 months, it can develop several months or years after an acute pulmonary embolism (APE) [8,9]. If a patient has signs and symptoms of pulmonary hypertension despite three months of anticoagulation following acute PE, CTEPH or CTED should be suspected.

A transthoracic echocardiogram and a six-minute walk test should be the initial diagnostic approach. If evidence of pulmonary hypertension is evident, the next step is a V/Q scan. If a mismatched segmental defect is noticed in the V/Q scan, computed tomographic angiography is done for further evaluation. If computed tomographic angiography shows suspicion of pulmonary hypertension, the next step is right heart catheterization with pulmonary angiogram +/- CT angiogram.

**Figure 4:**
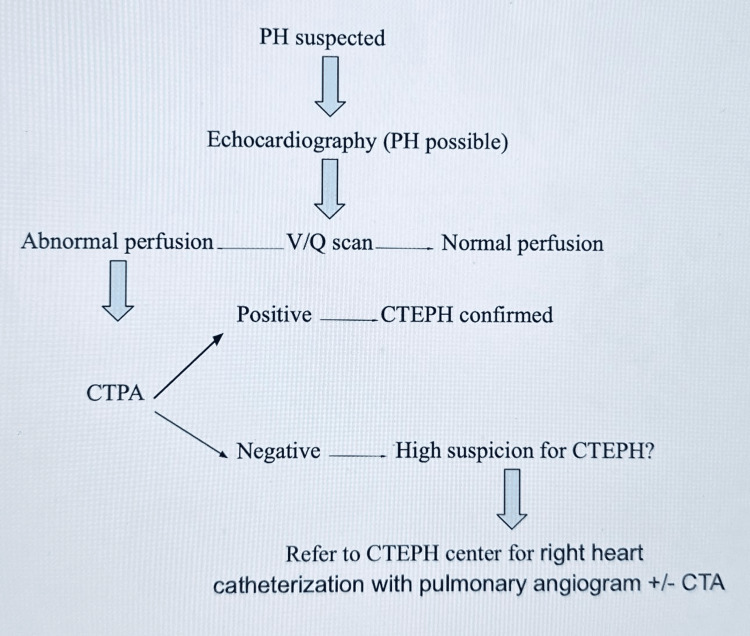
Algorithm of CTEPH diagnosis PH: pulmonary hypertension, V/Q: ventilation/perfusion, CTPA: computed tomographic pulmonary angiography, CTEPH: chronic thromboembolic pulmonary hypertension, CTA: computed tomography angiography.

Once the disease is confirmed, the patient should be assessed for operability [10]. If the patient is a candidate for surgery, pulmonary endarterectomy is the treatment of choice. Even though it is a complicated procedure, the quality of life of patients who undergo this procedure increases after the procedure. Pharmacological management +/- balloon angioplasty should be offered for a patient who is not a candidate for surgery. Balloon pulmonary angioplasty (BPA) also can be considered for patients who underwent endarterectomy with residual symptoms [2].

Pharmacological management consists of riociguat, treprostinil, bosentan, sildenafil, epoprostenol, anticoagulation therapy, furosemide, and oxygen therapy for heart failure and/or hypoxemia. Riociguat is a stimulator of soluble guanylate cyclase. A study showed that riociguat was effective and well-tolerated in patients of advanced age or risk factors for heart failure-reduced ejection fraction (HFrEF) [11]. Balloon pulmonary angioplasty (BPA) has shown some benefits in improvements both hemodynamically and functionally [12]. It also reduces the severity of continuing myocardial damage [13]. Early diagnosis and appropriate treatment of CTEPH can prevent the development of decompensated heart failure.

## Conclusions

CTEPH is a severe complication of PE. Diagnosis is quite challenging and causes delays in the treatment or worsening of the condition. Right heart failure in undiagnosed or untreated CTEPH increases mortality. Pulmonary endarterectomy is the preferred treatment of choice since the 6th World Symposium on PH. The patient undergoing surgery has both mortality and morbidity benefits. So early diagnosis and treatment of CTEPH are very important. A better understanding of pathophysiology and therapeutic targets will help in the development of new and more effective approaches for CTEPH.
